# P156: Ring wearing in healthcare settings: an evidence-based update

**DOI:** 10.1186/2047-2994-2-S1-P156

**Published:** 2013-06-20

**Authors:** A Dyar, OJ Dyar

**Affiliations:** 1 Oxford University, Oxford, UK

## Introduction

Tens of thousands of healthcare workers worldwide can only wear a plain wedding ring at work, if any at all. This arose from policies citing early laboratory evidence that rings can carry clinically relevant bacteria, but with little supporting clinical data. Policies that are both invasive and perceived as lacking evidence create a broader scepticism of infection control guidelines: it is therefore important to regularly review the evidence for such guidance.

## Methods

A systematic literature review was performed of studies investigating the infection risk of ring wearing by healthcare workers. PubMed, Cochrane Library and clinical trials registries were searched. Data was extracted on study design and quality, and the following outcomes: hospital acquired infection (HAI) rates, bacterial transmission, and bacterial contamination of healthcare workers’ hands.

## Results

Two interventional randomised controlled trials (RCTs) and ten observational studies were identified. No study investigated an association between ring wearing and HAI rates. The RCTs were very small and used hand colonization as the primary outcome. One RCT found higher colonization of hands of healthcare wokers randomised to wear rings than those not wearing rings, whereas the other RCT found no difference. One observational study assessed bacterial transmission through handshaking and found the presence of a ring did not result in higher transmission. Three observational studies found higher bacterial contamination of hands with rings, and five studies found no difference. The presence of rings did not result in higher contamination after handwashing in most studies. No study identified a significant increase in hand contamination with multiple rings compared with one ring, nor between different types of ring.

## Conclusion

No direct evidence was found that healthcare workers wearing rings results in higher HAI or bacterial transmission rates. Most studies did not identify higher contamination associated with ring wearing; furthermore, the clinical significance of a statistical difference in the number of colony forming units is unclear. Guidelines could benefit from reconsidering ring wearing guidance, and focussing on interventions with a more defined evidence base; fewer intrusions into healthcare workers’ personal autonomy may increase willingness to participate in other important interventions.

## Disclosure of interest

None declared

